# Human respiratory syncytial virus diversity and epidemiology among patients hospitalized with severe respiratory illness in South Africa, 2012–2015

**DOI:** 10.1111/irv.12905

**Published:** 2021-09-16

**Authors:** Ziyaad Valley‐Omar, Stefano Tempia, Orienka Hellferscee, Sibongile Walaza, Ebrahim Variava, Halima Dawood, Kathleen Kahn, Meredith McMorrow, Marthi Pretorius, Senzo Mtshali, Ernest Mamorobela, Nicole Wolter, Marietjie Venter, Anne von Gottberg, Cheryl Cohen, Florette K. Treurnicht

**Affiliations:** ^1^ National Institute for Communicable Diseases of the National Health Laboratory Service Johannesburg South Africa; ^2^ University of Cape Town Cape Town South Africa; ^3^ Centers for Disease Control and Prevention Pretoria South Africa; ^4^ Centers for Disease Control and Prevention Atlanta Georgia USA; ^5^ MassGenics Duluth Georgia USA; ^6^ University of the Witwatersrand Johannesburg South Africa; ^7^ University of KwaZulu‐Natal KwaZulu‐Natal South Africa; ^8^ Greys hospital Pietermaritzburg South Africa; ^9^ Novartis Pharma AG Basel Switzerland; ^10^ University of Pretoria Pretoria South Africa; ^11^ National Health Laboratory Service, Charlotte Maxeke Johannesburg Academic Hospital Johannesburg South Africa

**Keywords:** human respiratory syncytial virus, severe respiratory illness, South Africa

## Abstract

**Background:**

We aimed to describe the prevalence of human respiratory syncytial virus (HRSV) and evaluate associations between HRSV subgroups and/or genotypes and epidemiologic characteristics and clinical outcomes in patients hospitalized with severe respiratory illness (SRI).

**Methods:**

Between January 2012 and December 2015, we enrolled patients of all ages admitted to two South African hospitals with SRI in prospective hospital‐based syndromic surveillance. We collected respiratory specimens and clinical and epidemiological data. Unconditional random effect multivariable logistic regression was used to assess factors associated with HRSV infection.

**Results:**

HRSV was detected in 11.2% (772/6908) of enrolled patients of which 47.0% (363/772) were under the age of 6 months. There were no differences in clinical outcomes of HRSV subgroup A‐infected patients compared with HRSV subgroup B‐infected patients but among patients aged <5 years, children with HRSV subgroup A were more likely be coinfected with *Streptococcus pneumoniae* (23/208, 11.0% vs. 2/90, 2.0%; adjusted odds ratio 5.7). No significant associations of HRSV A genotypes NA1 and ON1 with specific clinical outcomes were observed.

**Conclusions:**

While HRSV subgroup and genotype dominance shifted between seasons, we showed similar genotype diversity as noted worldwide. We found no association between clinical outcomes and HRSV subgroups or genotypes.

## 1 BACKGROUND

In young children and infants, human respiratory syncytial virus (HRSV) is one of the predominant causes of acute respiratory tract illness. Globally, it was estimated that among children aged <5 years, 33.1 million episodes of HRSV‐associated severe acute respiratory illness (SARI) resulted in approximately 3.2 million (95% confidence interval [CI] 2.7–3.8 million) hospitalizations and 59,600 (95% CI 48,000–74,500) in‐hospital deaths in 2015; 99% of mortalities occurred in developing countries.^1^ Among South African patients admitted to sentinel surveillance hospitals for SARI, HRSV detection rates of 27% (1157/4293) and 4% (329/7796) were reported for children aged <5 years (2010–2011) and adults aged >18 years (2009–2013), respectively.^2^, ^3^ Within these populations, HIV coinfection was found to negatively affect disease outcomes including increased odds of hospitalization.^2^

HRSV is divided into two phylogenetically distinct subgroups (HRSV A and B), and these are further classified into multiple distinct genotypes based on sequence variability in the C‐terminus of the envelope glycoprotein G (G protein).^4^, ^5^, ^6^, ^7^ Since 2002, novel genotype BA, and since 2009 novel genotype ON1, displaying 60‐ and 72‐nucleotide sequence duplications (HRSV subgroups B and A, respectively) within the second variable domain of the C‐terminus have emerged.^4^, ^7^, ^8^, ^9^, ^10^, ^11^, ^12^, ^13^ To date, these novel genotypes have disseminated globally, becoming the dominant genotypes in circulation.^4^, ^7^, ^8^, ^9^, ^10^, ^14^ Contrasting findings have found no clear consensus between HRSV subgroups/genotypes and clinical outcomes.^4^, ^7^, ^8^, ^9^, ^10^, ^11^, ^12^, ^15^ Inconsistencies observed between studies may be due to small sample sizes and geographic variances in population diversity and herd immunity, which could predispose to or protect individuals from adverse clinical outcomes.

While no HRSV vaccines are currently available, global HRSV surveillance aided the development of multiple vaccine candidates that are in various stages of evaluation.^16^ The high degree of sequence variance and complex circulation patterns of HRSV resulted in challenges for development of an effective HRSV vaccine.^13^, ^16^, ^17^, ^18^, ^19^ These challenges include global and regional gaps in knowledge of HRSV epidemiology, subgroup and genotype geographic circulation patterns, target patient demographics, and outcomes for HRSV‐associated clinical illness.^16^ Regular updates of these knowledge gaps provide an information baseline that can be used to evaluate regional vaccine effectiveness when implemented and can inform vaccine formulation suitability and timing.

In this study, we aimed to describe the seasonal patterns and prevalence of HRSV subgroups and genotypes among hospitalized patients with severe respiratory illness (SRI) in South Africa, from 2012 through 2015. We also aimed to compare the associations of HRSV subgroups and genotypes with epidemiologic characteristics and clinical outcomes.

## 2 METHODS

### 2.1 Study design and population

We enrolled participants in a prospective hospital‐based SRI surveillance program from 01 January 2012 through 31 December 2015 at Edendale and Klerksdorp‐Tshepong Hospitals situated in periurban areas in the subtropical KwaZulu‐Natal and temperate North West Provinces of South Africa, respectively. The SRI case definition included hospitalized individuals of any age with illness onset of any duration prior to admission meeting age‐specific inclusion criteria. Children aged 2 days to <3 months included any hospitalized patient with diagnosis of suspected sepsis or physician‐diagnosed lower respiratory tract infection.^20^ Children aged 3 months to <5 years included any hospitalized patient with physician‐diagnosed lower respiratory tract infection, including bronchitis, bronchiolitis, pneumonia, and pleural effusion.^20^ Individuals aged ≥5 years included any hospitalized patient presenting with lower respiratory tract infection with temperature ≥38°C or history of fever and cough.^20^ A case investigation form that included clinical details (fever [≥38°C], cough, requirement for supplemental oxygen, prolonged hospitalization [≥5 days], in‐hospital death, admission to ICU, tachypnea, stridor) and underlying medical conditions (HIV infection, prematurity, heart disease, malnutrition, chronic lung disease, and asthma) was completed for all enrolled patients.

### 2.2 Sample collection

Respiratory samples (nasopharyngeal aspirates for children <5 years of age and combined nasopharyngeal and oropharyngeal swabs from individuals ≥5 years of age) were collected and placed in universal transport medium (Copan, California, USA), stored at 4°C–8°C and transported within 72 h of collection to the National Institute for Communicable Diseases (NICD), Johannesburg, South Africa, for testing.^20^ Whole blood samples (EDTA) were collected from consenting patients for the detection of *Streptococcus pneumoniae* (*S. pneumoniae*) by *lytA* polymerase chain reaction (PCR).

### 2.3 Determination of HIV status

HIV infection status was obtained from a combination of two sources: (i) patient clinical records and (ii) for consenting patients, dried blood spots that were tested by enzyme‐linked immunosorbent assay (ELISA) for patients aged ≥18 months and PCR for children aged <18 months if the ELISA was reactive or exposure status was unknown.

### 2.4 Laboratory detection of HRSV

Samples collected from 2012 through 2014 were tested for the presence of HRSV and other respiratory viruses (influenza A and B viruses, parainfluenza virus [PIV] types 1–3, adenoviruses, rhinoviruses, human metapneumovirus [hMPV], and enteroviruses) using an in‐house multiplex real‐time reverse transcription PCR (rt‐RTPCR) assay.^21^ From 2015, a validated commercial one‐step multiplex rt‐RTPCR assay, FTD® Flu‐HRSV kit (FastTrack Diagnostics, Luxembourg), was used for the detection of HRSV and influenza A and B viruses. The 2015 samples were also tested for adenoviruses, enteroviruses, parainfluenza viruses (PIV) types 1–4, human metapneumovirus (hMPV), and rhinoviruses as before but now human bocaviruses and seasonal human coronaviruses types (NL63, 229E and OC43) were included in our testing panel using the Allplex one‐step rt‐RTPCR respiratory panels 2 and 3 (Seegene, Seoul, Korea).

### 2.5 Determination of HRSV A/B subgroup by real‐time reverse transcription PCR

HRSV subgroup was determined on all subgrouped samples using an in‐house one‐step rt‐RTPCR designed for the detection of HRSV A and HRSV B using previously published methods.^22^, ^23^

### 2.6 Amplification and sequencing of the HRSV G protein gene

The complete HRSV G‐protein gene was PCR‐amplified following random primer cDNA synthesis using the SuperScript III reverse transcriptase kit (Invitrogen, Life Technologies, Carlsbad, CA, USA). A first round PCR utilized 5 μl of cDNA, the G1‐21^14^ and F164 primers^24^ with Platinum *Pfx* DNA polymerase system (Invitrogen, Life Technologies). The second round PCR utilized the G32A(HRSV A), G598A(HRSV A) /G32B(HRSV B), G604B(HRSV B) forward, or G665R and F1 reverse primers as described.^25^ HRSV‐positive samples with insufficient volume or positives with cycle threshold (Ct)‐values of >35 were not PCR amplified for sequencing as they were unlikely to successfully amplify. PCR products were analyzed and viewed following gel electrophoresis on a 1% agarose gel and purified using the Wizard® SV Gel and PCR Clean‐Up System (Promega Corporation, Madison, WI, USA). Cycle sequencing was performed with the BigDye terminator 3.1 cycle sequencing kit (Applied Biosystems, Foster City, CA, USA). Sequences were assembled using Sequencher® version 5 (Gene Codes Corporation, Michigan, USA). Sequences with the following accession numbers MN516831 to MN517111 were uploaded to GenBank.

### 2.7 Phylogenetic analysis of HRSV partial G protein genes

Unique HRSV A and HRSV B virus G‐gene nucleotide sequences representing the second variable domain (330 base pairs: nucleotides 5323–5652) were aligned separately using Multiple Sequence Comparison by Log Expectation (MUSCLE), along with unique international genotype reference sequences (accession numbers shown in Figure 3), downloaded from the GenBank sequence database, using default settings.^26^ As the GenBank database (https://www.ncbi.nlm.nih.gov/genbank/) predominantly contains partial HRSV G protein gene sequences spanning the second variable domain; this was the gene region selected for partial sequence G‐protein alignments in this analysis. Phylogenies were determined by the construction of maximum likelihood phylogenetic trees using RaxML (Heidelberg Institute for Theoretical Studies, Heidelberg, Germany) with the GTR‐GAMMA nucleotide substitution model with branch support assessed with 100 bootstrap replicates.^27^

### 2.8 Statistical analysis

Unconditional logistic regression was used to assess factors associated with HRSV‐positivity and factors associated with HRSV A and HRSV B‐positivity among individuals hospitalized with SRI aged <5 years and ≥5 years separately. We assessed factors associated with commonly identified genotypes, ON1 and NA1 among HRSV‐positive patients aged <5 years with available genotype results. We further assessed factors associated with presence of fever among HRSV‐positive patients of all ages. A random effect on admission facility was included for all analysis to account for potential differences in the service population. Variables for which the *p* value in univariate analysis was ≤0.2 were assessed in multivariable models by forward selection and statistical significance was assessed at *p* < 0.05. The analysis was performed using STATA 14.1 (StataCorp, College Station, TX, USA).

### 2.9 Ethical considerations

The SRI surveillance protocol was reviewed and approved by the University of the Witwatersrand Human Research Ethics Committee (HREC; protocol number M081042), the University of KwaZulu Natal, Human Biomedical Research Ethics Committee (BREC) protocol number BF157/08. This surveillance protocol was reviewed and deemed nonresearch (number 2012‐6197) by the US Centers for Disease Control and Prevention (CDC, Atlanta, Georgia, USA).

## 3 RESULTS

### 3.1 Study population

From 2012 through 2015, we enrolled 6910 patients with SRI of which 6908 (>99.9%) had available laboratory test results and were included in the analysis. Of these, 36.3% (2509/6908) were children aged <5 years (Table 1) and 49.6% (3421/6897) were female. HIV results were available for 94.1% (6498/6908) of patients of whom 51.9% (3372/6498) were HIV infected. The HIV prevalence was lowest (10.1%; 143/1417) among infants aged <1 year and highest (90.5%; 1884/2081) among individuals aged 25–44 years.

**TABLE 1:** Factors associated with HRSV infection among children aged <5 years, hospitalized with severe respiratory illness, Edendale and Klerksdorp‐Tshepong hospitals, South Africa, 2012–2015

Characteristic	HRSV positive *n*/*N* (%)	HRSV negative *n*/*N* (%)	Univariate odds ratio (95% CI)	*p* value	Multivariable adjusted odds ratio (95% CI) ≤ 5 years	*p* value
Total	601/2509 (23.9)	1908/2509 (76.1)				
Age						
<3 months	229/601 (38.1)	360/1908 (18.9)	4.8 (3.5–6.7)	<0.001	4.5 (3.0–6.9)	**<0.001**
3–5 months	134/601 (22.3)	306/1908 (16.0)	3.3 (2.4–4.7)	<0.001	3.4 (2.2–5.2)	**<0.001**
6–11 months	106/601 (17.6)	404/1908 (21.2)	2.0 (1.4–2.8)	<0.001	2.0 (1.3–3.1)	**0.001**
12–23 months	78/601 (13.0)	427/1908 (22.4)	1.4 (1.0–2.0)	0.083	1.6 (1.1–2.6)	**0.024**
24–59 months	54/601 (9.0)	411/1908 (21.5)	Reference		Reference	‐
Female	256/600 (42.7)	844/1907 (44.3)	0.9 (0.8–1.1)	0.493		
Year						
2012	188/601 (31.3)	521/1908 (27.3)	Reference			
2013	165/601 (27.5)	590/1908 (30.9)	0.8 (0.6–0.9)	0.037		
2014	133/601 (22.1)	401/1908 (21.0)	0.9 (0.7–1.2)	0.521		
2015	115/601 (19.1)	396/1908 (20.8)	0.8 (0.6–1.1)	0.110		
Clinical presentation and course						
Fever ≥38°C	370/598 (61.9)	1219/1896 (64.3)	0.9 (0.7–1.1)	0.284	1.3 (1.0–1.7)	**0.035**
Cough	581/596 (97.5)	1615/1890 (85.4)	6.6 (3.9–11.2)	<0.001	7.6 (4.2–13.7)	**<0.001**
Supplemental oxygen needed	409/599 (68.3)	1008/1889 (53.4)	1.9 (1.5–2.3)	<0.001	1.4 (1.1–1.8)	**0.002**
Prolonged hospitalization (≥5 days)	302/601 (50.2)	913/1908 (47.9)	1.1 (0.9–1.3)	0.305		
In‐hospital death	4/597 (0.7)	29/1883 (1.54)	0.4 (0.1–1.2)	0.082		
Admitted to ICU	24/598 (4.0)	80/1887 (4.2)	0.9 (0.6–1.5)	0.809	0.4 (0.2–0.8)	**0.012**
Tachypnea	335/595 (56.3)	879/1880 (46.8)	1.5 (1.2–1.8)	<0.001		
Stridor	121/595 (20.3)	324/1878 (17.3)	1.2 (0.9–1.5)	0.091		
Underlying medical conditions						
HIV infection	32/549 (5.8)	246/1756 (14.0)	0.4 (0.3–0.6)	<0.001	0.4 (0.3–0.6)	**<0.001**
Prematurity	68/600 (11.3)	257/1907 (13.5)	0.8 (0.6–1.1)	0.167		
Heart disease	1/600 (0.2)	5/1906 (0.3)	0.6 (0.07–5.44)	0.663		
Malnutrition	92/534 (17.2)	469/1746 (26.9)	0.6 (0.4–0.7)	<0.001	0.5 (0.4–0.7)	**<0.001**
Chronic lung disease	1/600 (0.2)	3/1905 (0.2)	1.1 (0.11–10.2)	0.961		
Asthma	7/600 (1.2)	27/1906 (1.4)	0.8 (0.4–1.9)	0.639		
Coinfection						
Tuberculosis	6/150 (4)	24/559 (4.3)	0.9 (0.4–2.3)	0.873		
*Streptococcus pneumoniae*	35/404 (8.7)	151/1348 (11.2)	0.8 (0.5–1.1)	0.138		
Influenza A/B	10/601 (1.7)	130/1908 (6.8)	0.2 (0.1–0.4)	<0.001	0.2 (0.1–0.4)	**<0.001**
Rhinovirus	154/601 (25.6)	763/1908 (40.0)	0.5 (0.4–0.6)	<0.001	0.4 (0.3–0.5)	**<0.001**
Adenovirus	93/601 (15.5)	460/1908 (24.1)	0.6 (0.5–0.7)	<0.001		
Enterovirus	35/601 (5.8)	142/1908 (7.4)	0.8 (0.5–1.1)	0.168	0.6 (0.4–0.9)	
Human metapneumovirus	5/601 (0.8)	115/1908 (6.0)	0.1 (0.05–0.3)	<0.001	0.1 (0.04–0.3)	**<0.001**
Parainfluenza virus types 1, 2, 3	10/601 (1.7)	168/1908 (8.8)	0.2 (0.1–0.3)	<0.001	0.2 (0.1–0.5)	**0.003**

*Note*: Bold emphasizes statistically significant variables.

CI = confidence interval; ICU = intensive care unit; *n*/*N* = sample size/population size.

### 3.2 HRSV detection

**FIGURE 1:**
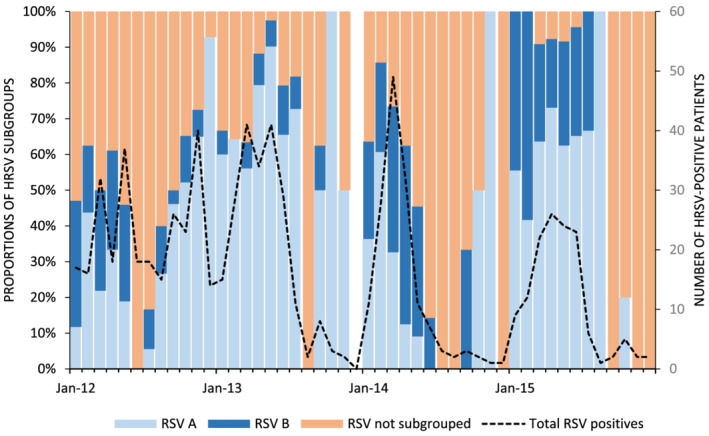
Monthly number of human respiratory syncytial virus (HRSV)‐positive patients hospitalized with severe respiratory illness, Edendale and Klerksdorp‐Tshepong hospitals, South Africa, 2012–2015. The proportion of positives belonging to subgroup A, B and not typed is also indicated in the bar graph

During the study period, HRSV was detected in 11.2% (772/6908) of samples, 24.0% (601/2509) among individuals aged <5 years and 3.9% (171/4399) among individuals ≥5 years of age. Of the 772 HRSV‐infected patients, 47.0%, (363/772) were <6 months of age. Year‐round HRSV activity was observed across the 4‐year observation period with a surge of HRSV activity observed each year between March and May (autumn) (2012–2015) resulting in a single peak per year (Figure 1). An apparent second peak or surge of HRSV activity was noted between September and December of 2012 (Spring–Summer). This second peak was only observed for patients admitted to Edendale Hospital located in the KwaZulu‐Natal Province of South Africa (Figure S1).

### 3.3 Factors associated with HRSV‐positivity in patients hospitalized with SRI

On multivariable analysis considering clinical presentation, underlying medical conditions and coinfection variables among persons aged <5 years, a higher odds of HRSV infection was associated with younger age groups when compared to individuals aged 24–59 months (<3 months adjusted odds ratio [aOR]: 4.5; 3–5 months aOR: 3.4; 6–11 months aOR: 2.0; 12–23 months aOR: 1.6). In addition, HRSV‐positive children were more likely to present with temperature ≥38°C (aOR: 1.3), have a cough (aOR: 7.6), and receive oxygen support during admission (aOR: 1.4) (Table 1). HRSV‐positive children were less likely to be admitted to the intensive care unit (ICU) (aOR: 0.4), to be malnourished (aOR: 0.5), be HIV infected (aOR: 0.4), and be coinfected with influenza viruses (aOR: 0.2), rhinoviruses (aOR: 0.4), enteroviruses (aOR: 0.6), hMPV (aOR: 0.1), and PIV 1–3 (aOR: 0.2) than HRSV‐negative children (Table 1). HRSV positive persons aged <5 years, persons aged ≥3 months were more likely to present with fever when compared to those <3 months (3–5 months aOR: 2.2, 95% CI: 1.7–2.8; 6–11 months aOR: 2.9, 95% CI: 2.3–3.8; 12–23 months aOR: 3.2, 95% CI: 2.4–4.1; 24–59 months aOR: 2.8, 95% CI: 2.2–3.7) (data not shown).

HRSV‐positive individuals ≥5 years were more likely to be HIV infected (aOR: 1.6) and to be coinfected with enteroviruses (aOR: 7.3) than HRSV‐negatives children aged <5 years (Table 2).

**TABLE 2:** Factors associated with HRSV infection among patients aged ≥5 years, hospitalized with severe respiratory illness, Edendale and Klerksdorp‐Tshepong hospitals, South Africa, 2012–2015

Characteristic	HRSV positive *n*/*N* (%) (*N* = 171)	HRSV negative *n*/*N* (%)	Univariate odds ratio (95% CI)	*p* value	Multivariable adjusted odds ratio (95% CI) years	*p* value
Total	171/4399 (3.9)	4228/4399 (96.1)				
Age						
5–24 years	38/171 (22.2)	562/4228 (13.3)	Reference		Reference	
25–44 years	82/171 (48.0)	2094/4228 (49.5)	0.6 (0.4–0.9)	0.007	0.6 (0.4–0.9)	**0.008**
45–64 years	39/171 (22.8)	1226/4228 (29.0)	0.5 (0.3–0.7)	0.001	0.5 (0.3–0.9)	**0.010**
65 + years	12/171 (7.0)	346/4228 (8.2)	0.5 (0.3–0.9)	0.048	0.8 (0.4–1.7)	0.606
Female	102/170 (60)	2219/4220 (52.6)	1.4 (0.9–1.8)	0.060		
Year						
2012	86/171 (50.3)	1487/4230 (35.2)	Reference		Reference	
2013	49/171 (28.7)	1128/4230 (26.7)	0.8 (0.5–1.1)	0.118	0.8 (0.5–1.1)	0.160
2014	17/171 (9.9)	836/4230 (19.8)	0.4 (0.2–0.6)	<0.001	0.4 (0.2–0.6)	**<0.001**
2015	19/171 (11.1)	779/4230 (18.4)	0.4 (0.3–0.7)	0.001	0.5 (0.3–0.8)	**0.009**
Clinical presentation and course						
Fever ≥38°C	69/169 (40.8)	1688/4197 (40.2)	1.0 (0.8–1.4)	0.874		
Cough	128/148 (86.5)	3503/3833 (91.4) 333	0.6 (0.4–0.9)	0.053		
Supplemental oxygen needed	74/170 (43.5)	1562/4147 (37.7)	1.3 (0.9–1.7)	0.126		
Prolonged hospitalization (≥5 days)	117/171 (68.4)	2848/4230 (67.3)	1.1 (0.8–1.5)	0.765		
In‐hospital death	19/162 (11.7)	485/4106 (11.8)	0.9 (0.6–1.6)	0.974		
Admitted to ICU	1/169 (0.6)	21/4144 (0.5)	1.2 (0.2–8.7)	0.882		
Tachypnea	2/4 (50)	6/22 (27.3)	2.7 (0.3–23.4)	0.380		
Stridor	0/4 (0)	3/22 (13.6)				
Underlying medical conditions						
HIV infection	125/156 (80.1)	2943/3966 (74.2)	1.4 (0.9–2.1)	0.088	1.6 (1.0–2.6)	**0.033**
Heart disease	3/170 (1.8)	72/4222 (1.7)	1.0 (0.3–3.3)	0.954		
Chronic lung disease	0/170 (0)	28/4222 (0.7)				
Asthma	7/170 (4.1)	142/4222 (3.4)	1.2 (0.6–2.7)	0.606		
Coinfection						
Tuberculosis	18/101 (17.8)	653/2706 (24.1)	0.7 (0.4–1.1)	0.132		
*Streptococcus pneumoniae*	21/162 (13.0)	465/3965 (11.7)	1.1 (0.7–1.8)	0.637		
Influenza	7/171 (4.1)	185/4230 (4.4)	0.9 (0.4–2.0)	0.859		
Rhinovirus	33/171 (19.3)	693/4230 (16.4)	1.2 (0.8–1.8)	0.324		
Adenovirus	27/171 (15.8)	296/4230 (7.0)	2.5 (1.6–3.8)	<0.001		
Enterovirus	10/171 (5.8)	35/4230 (0.8)	7.4 (3.6–15.3)	<0.001	7.3 (3.4–15.7)	**<0.001**
Human metapneumovirus	2/171 (1.2)	46/4230 (1.1)	1.1 (0.3–4.5)	0.920		
Parainfluenza virus types 1, 2, 3	1/171 (0.6)	86/4230 (2.0)	0.3 (0.04–2.04)	0.119		

*Note*: Bold emphasizes statistically significant variables.

CI = confidence interval; ICU = intensive care unit; *n*/*N* = sample size/population size.

### 3.4 HRSV subgroup detection and factors associated with HRSV subgroups among HRSV‐positive patients hospitalized with SRI

About two thirds, 67.2% (519/772) of HRSV‐positive samples, were subgrouped, of which 71.1% (369/519) were HRSV A. HRSV A was the predominate subgroup across all years except 2014 (2012: 68.8%, 2013: 90.3%, and 2015: 67.5%) (Figure 1). Similar proportions of HRSV A (45.8%, 44/96) and HRSV B (54.2%, 52/96) were seen in 2014. HRSV A positive patients <5 years were more likely to be coinfected with *S. pneumoniae* (aOR: 5.7), but there were no clinical differences (Table 3). No statistically significant differences were found between HRSV A and HRSV B positive individuals aged ≥5 years (Table S1).

**TABLE 3:** Factors associated with HRSV subgroups among children aged <5 years, hospitalized with severe respiratory illness, Edendale and Klerksdorp‐Tshepong hospitals, South Africa, 2012–2015

		HRSV A subgroup	HRSV B subgroup				
Characteristic	HRSV subgrouped *n*/*N* (%)	Samples positive/samples tested (%)	Samples positive/samples tested (%)	Univariate odds ratio (95% CI)	*p* value	Multivariable adjusted odds ratio (95% CI) < 5 years	*p* value
Total	449/601 (74.7)	319/449 (71.0)	130/449 (29.0)				
Age							
<3 months	165/449 (36.7)	115/319 (36.1)	50/130 (38.5)	Reference			
3–5 months	105/449 (23.4)	76/319 (23.8)	29/130 (22.3)	1.1 (0.7–1.9)	0.637		
6–11 months	77/449 (17.1)	60/319 (18.8)	17/130 (13.1)	1.5 (0.8–2.9)	0.185		
12–23 months	52/449 (11.6)	44/319 (13.8)	18/130 (13.8)	1.1 (0.6–2.0)	0.852		
24–59 months	40/449 (8.9)	24/319 (7.5)	16/130 (12.3)	0.7 (0.3–1.3)	0.241		
Female	198/449 (44.1)	137/319 (42.9)	61/130 (46.9)	0.9 (0.6–1.3)	0.442		
Year							
2012	122/449 (27.2)	87/319 (27.3)	35/130 (26.9)	Reference		Reference	
2013	139/449 (31.0)	125/319 (39.2)	14/130 (10.8)	3.6 (1.8–7.1)	<0.001	5.2 (2.0–13.3)	**0.001**
2014	86/449 (19.2)	40/319 (12.5)	46/130 (35.4)	0.3 (0.2–0.6)	<0.001	0.5 (0.2–0.9)	**0.027**
2015	102/449 (22.7)	67/319 (21.0)	35/130 (26.9)	0.8 (0.4–1.4)	0.366	0.8 (0.4–1.6)	0.535
Clinical presentation and course							
Fever ≥38°C	275/448 (61.4)	192/319 (60.2)	83/129 (64.3)	0.8 (0.5–1.3)	0.412		
Cough	439/447 (98.2)	311/317 (98.1)	128/130 (98.5)	0.8 (0.2–4.1)	0.795		
Supplemental oxygen needed	310/449 (69.0)	222/319 (69.6)	88/130 (67.7)	1.1 (0.7–1.7)	0.694		
Prolonged hospitalization (≥5 days)	226/449 (50.3)	162/319 (50.8)	64/130 (49.2)	1.1 (0.7–1.6)	0.765		
In‐hospital death	3/448 (0.7)	3/318 (0.9)	0/130 (0)				
Admitted to ICU	16/449 (3.6)	8/319 (2.5)	8/130 (6.2)	0.4 (0.1–1.1)	0.072		
Tachypnea	249/447 (55.7)	173/317 (54.6)	76/130 (58.5)	0.9 (0.6–1.3)	0.452		
Stridor	88/447 (19.7)	63/317 (19.9)	25/130 (19.2)	1.0 (0.6–1.7)	0.876		
Underlying medical conditions							
HIV infection	21/417 (5.0)	12/297 (4.0)	9/120 (7.5)	0.5 (0.2–1.3)	0.158		
Prematurity	52/449 (11.6)	40/319 (12.5)	12/130 (9.2)	1.4 (0.7–2.8)	0.311		
Heart disease	1/449 (0.2)	0/319 (0)	1/130 (0.8)				
Malnutrition	70/405 (17.3)	52/297 (17.5)	18/108 (16.7)	1.1 (0.6–1.9)	0.843		
Chronic lung disease	1/449 (0.22)	0/319 (0)	1/130 (0.8)				
Asthma	3/449 (0.7)	3/319 (0.9)	0/130 (0)				
Coinfection							
Tuberculosis	4/121 (3.3)	4/93 (4.3)	0/28 (0)				
*Streptococcus pneumoniae*	25/298 (8.4)	23/208 (11.1)	2/90 (2.2)	5.5 (1.3–23.7)	0.005	5.7 (1.3–25.6)	**0.024**
Influenza	7/449 (1.6)	5/319 (1.6)	2/130 (1.5)	1.0 (0.2–5.3)	0.982		
Rhinovirus	113/449 (25.2)	79/319 (24.8)	34/130 (26.2)	0.9 (0.6–1.5)	0.759		
Adenovirus	64/449 (14.3)	48/319 (15.0)	16/130 (12.3)	1.3 (0.7–2.3)	0.446		
Enterovirus	20/449 (4.5)	16/319 (5.0)	4/130 (3.1)	1.7 (0.5–5.1)	0.350		
Human metapneumovirus	5/449 (1.1)	5/319 (1.6)	0/130 (0)				
Parainfluenza virus types 1, 2, 3	8/449 (1.8)	7/319 (2.2)	1/130 (0.8)	2.9 (0.4–23.8)	0.263		

*Note*: Bold emphasizes statistically significant variables.

CI = confidence interval; ICU = intensive care unit; *n*/*N* = sample size/population size.

### 3.5 HRSV genotype prevalence and factors associated with HRSV genotypes among HRSV‐positive patients hospitalized with SRI

Among the 519 HRSV A/B‐subgrouped specimens, 33.7% (175/519) of HRSV positives (A: 82.3% [144/175]; B: 17.7% [31/175]) were sequenced and included for phylogenetic analysis. A total of 18.7% (144/519) and 30.1% (156/519) were excluded from G‐protein amplification and sequencing due to low nucleic acid concentration (Ct value >35) and insufficient specimen volume, respectively, whereas 8.5% (44/519) of specimens yielded low quality, unusable sequence data.

**FIGURE 2:**
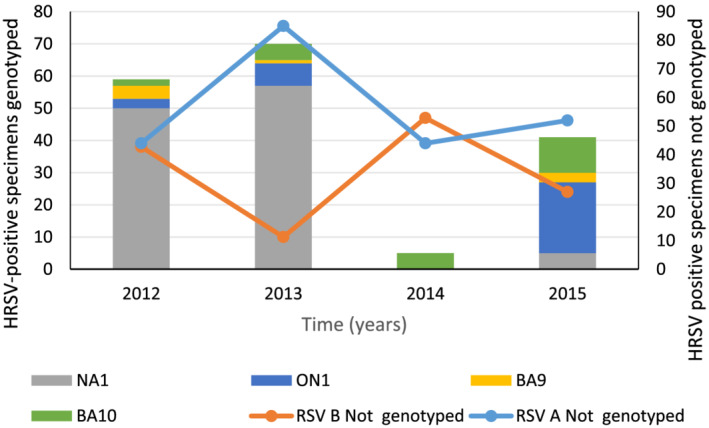
Human respiratory syncytial virus (HRSV) genotypes detected among patients of any age, hospitalized with severe respiratory illness, Edendale and Klerksdorp‐Tshepong hospitals, South Africa, 2012–2015

**FIGURE 3:**
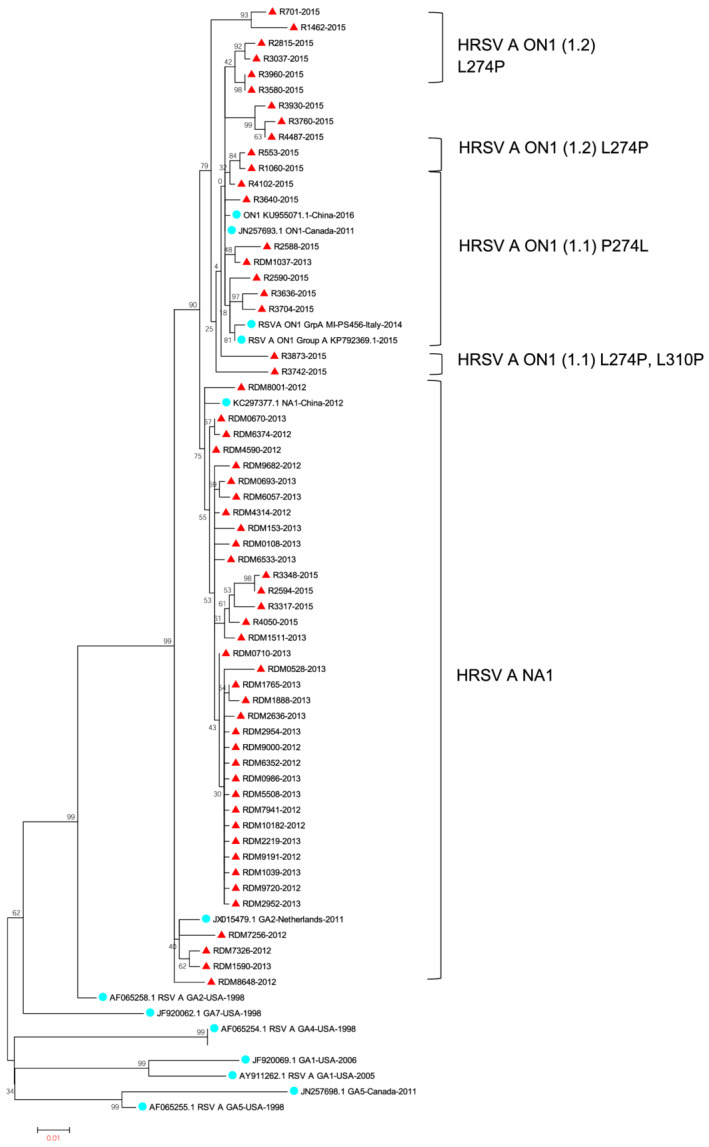
Inferred phylogeny of human respiratory syncytial virus (HRSV) A G‐gene second variable domain of south African HRSV A isolates (red triangles) based on maximum likelihood model (Mega 5.0 under GTR GAMMA model of nucleotide evolution). Selected publicly available sequences global HRSV A sequences are represented by GenBank accession numbers (blue circles). Brackets indicate HRSV NA1 and ON1 (1.1, 1.2, and 1.3) lineages. Sample dates indicated by last 4 digits of sample names

The HRSV A genotype NA1 predominated in 2012 (94% [50/53]) and 2013 (89% [57/64]), and ON1 in 2015 (81% [22/27]) (Figure 2). HRSV B genotypes BA9 and BA10 circulated concurrently with HRSV A genotypes in 2012–2015. Five HRSV B positives genotyped in 2014 were all genotype BA10. HRSV A ON1 lineages 1.1, 1.2, and 1.3 were circulating in 2015 (Figure 3); 77% (135/175) of samples genotyped were from the <5 years age category. In this age category, HRSV A genotypes NA1 and ON1 showed associations with year and prolonged hospitalization (≥5 days) but these did not remain significant in the multivariable model (Table 4).

**TABLE 4:** Factors associated with HRSV A genotypes among patients aged <5 years, hospitalized with severe respiratory illness, Edendale and Klerksdorp‐Tshepong hospitals, South Africa, 2012–2015

		NA1 genotype	ON1 genotype		
Characteristic	HRSV genotyped *n*/*N* (%)	Samples positive/samples tested (%)	Samples positive/samples tested (%)	Univariate odds ratio (95% CI)	*p* value
Total	135/319 (42.3)	104/135 (77.0)	31/135 (23.0)		
Age					
<3 months	53/135 (39.3)	41/104 (39.4)	12/31 (38.7)	Reference	
3–5 months	35/135 (25.9)	28/104 (26.9)	7/31 (22.6)	0.8 (0.3–2.4)	0.768
6–11 months	23/135 (17.0)	18/104 (17.3)	5/31 (16.1)	0.9 (0.3–3.1)	0.931
12–23 months	14/135 (10.4)	10/104 (9.6)	4/31 (12.9)	1.4 (0.4–5.2)	0.644
24–59 months	10/135 (7.4)	7/104 (6.7)	3/31 (9.7)	1.5 (0.3–6.6)	0.618
Female	62/135 (45.9)	52/104 (50.0)	10/31 (32.3)	0.5 (0.2–1.1)	0.079
Year					
2012	50/135 (37.0)	47/104 (45.2)	3/31 (9.7)	Reference	
2013	61/135 (45.2)	54/104 (51.9)	7/31 (22.6)	2.0 (0.5–8.3)	0.324
2015	24/135 (17.8)	3/104 (2.9)	21/31 (67.7)	109.7 (20.4–588.9)	<0.001
Clinical presentation and course					
Fever ≥38°C	82/135 (60.7)	63/104 (60.6)	19/31 (61.3)	1.0 (0.5–2.3)	0.943
Cough	132/134 (98.5)	102/103 (99.0)	30/31 (96.8)	0.3 (0.2–4.8)	0.405
Supplemental oxygen needed	91/134 (67.9)	72/103 (69.9)	19/31 (61.3)	0.7 (0.3–1.6)	0.373
Prolonged hospitalization (≥5 days)	73/135 (54.1)	62/104 (59.6)	11/31 (35.5)	0.4 (0.2–0.9)	0.018
In‐hospital death	2/133 (1.5)	1/102 (0.98)	1/31 (3.2)	3.4 (0.2–55.5)	0.408
Admitted to ICU	1/134 (0.75)	1/103 (0.97)	0/31 (0)		
Tachypnea	79/134 (59.0)	60/103 (58.3)	19/31 (61.3)	1.1 (0.5–2.6)	0.762
Stridor	29/134 (21.6)	26/103 (25.2)	3/31 (9.7)	0.3 (0.1–1.1)	0.049
Underlying medical conditions					
HIV infection	5/123 (4.1)	5/94 (5.3)	0/29 (0)		
Prematurity	14/135 (10.4)	12/104 (11.5)	2/31 (6.5)	0.5 (0.1–2.5)	0.392
Heart disease	0/135 (0)				
Malnutrition	25/132 (18.9)	21/101 (20.8)	4/31 (12.9)	0.6 (0.2–1.8)	0.311
Chronic lung disease	0/135 (0)				
Asthma	1/135 (0.7)	0/104 (0)	1/31 (3.2)		
Coinfection					
Tuberculosis	1/35 (2.9)	1/27 (3.7)	0/8 (0)		
*Streptococcus pneumoniae*	8/83 (9.6)	6/66 (9.1)	2/17 (11.8)	1.3 (0.2–7.3)	0.745
Influenza	1/135 (0.7)	1/104 (1.0)	0/31 (0)		
Rhinovirus	39/135 (28.9)	32/104 (30.8)	7/31 (22.6)	0.7 (0.3–1.7)	0.369
Adenovirus	22/135 (16.3)	21/104 (20.2)	1/31 (3.2)	0.1 (0.02–1.02)	0.010
Enterovirus	5/135 (3.7)	4/104 (3.8)	1/31 (3.2)	0.8 (0.1–7.7)	0.871
Human metapneumovirus	1/135 (0.7)	1/104 (1.0)	0/31 (0)		
Parainfluenza virus types 1, 2, 3	1/135 (0.7)	0/104 (0)	1/31 (3.2)		

*Note*: CI = confidence interval; ICU = intensive care unit; *n*/*N* = sample size/population size.

## 4 DISCUSSION

Similar to previous studies conducted in South Africa and other temperate climates, low levels of HRSV activity occurred year‐round in individuals admitted for SRI over the 4‐year surveillance period with peaks of activity occurring between the autumn and early winter months.^2^, ^3^, ^28^, ^29^ In 2012, higher year‐round as well as an additional peak of HRSV activity during the September–December period (Spring–Summer) was observed at Edendale Hospital, which is in the subtropical climate of the KwaZulu‐Natal Province. This is consistent with previous observations in tropical and subtropical climates, which demonstrate year round activity with some seasonal peaks, which may not be consistent over consecutive years.^29^

In patients with SRI, prevalence of HRSV‐associated illness was 23.9% (601/2509) and 3.9% (171/4399), respectively, in patients aged <5 and ≥5 years showing trends consistent with previous South African SARI studies (27% and 4% prevalence in children [<5 years] and adults [>18 years], respectively^2^, ^3^). Among children aged <5 years, HRSV‐infected patients were more likely to have fever (≥38° C), cough, and the requirement for supplemental oxygen but were less likely to be malnourished, HIV‐infected, admitted to ICU, or have respiratory virus coinfections. Within the <5 years age group, we also showed that HRSV‐infected children aged ≥3 months were more likely to present with fever when compared to children aged <3 months. This corroborates previous findings that inclusion of fever in the SARI/SRI case definition could limit HRSV case detection among young children hospitalized with lower respiratory tract infection.^30^ Moderate to late preterm infants born at 33–35 weeks gestational age have previously also been shown to require supplemental oxygen during HRSV infection and admission to the ICU.^31^ The decreased likelihood of ICU admission for HRSV‐positive patients aged <5 years observed in this study seems counterintuitive but studies that have shown an increased likelihood have done so in populations with a high prevalence of underlying disorders.^32^ Previous studies from South Africa reported that respiratory virus coinfection in children aged <5 years with HRSV‐associated SRI was unlikely.^33^, ^34^ Individuals in the ≥5‐year age group with HRSV‐associated SRI, on the contrary, were more likely to be HIV infected, consistent with higher HIV prevalence in these age groups. As both humoral and cellular immunity are required to control acute HRSV infections, an increased likelihood of HRSV infection in ≥5 year HIV‐infected individuals may stem from an HIV‐directed impairment of host immunity, while in the <5 year age group, it is likely that HRSV infection is driven by HRSV‐naïve immune systems.^35^, ^36^, ^37^ Moyes et al. showed that HRSV‐infected patients (<5 years) with SARI who were HIV‐infected demonstrate a threefold to fivefold increased odds of hospitalization, higher odds of death, and increased length of hospitalization when compared with HIV‐negative children.^2^

Similar to previous studies, we demonstrated dynamic HRSV subgroup prevalence characterized by the cocirculation and year‐on‐year changes in the prevalence of each subgroup but favoring HRSV A (2012: 69%, 2013: 90%, 2014: 46%, and 2015: 68%) as the most dominant subgroup in most years. While some studies have shown a trend toward HRSV A causing more severe clinical outcomes when compared with HRSV B, there is currently no consensus.^15^, ^38^, ^39^, ^40^, ^41^, ^42^, ^43^, ^44^, ^45^ In our study, most clinical presentations or outcomes, underlying medical conditions, and coinfection variables demonstrated no statistically significant association with the detection of HRSV subgroups in patients. In our study, persons aged <5 years with HRSV A‐related illness were more likely be coinfected with *S. pneumoniae* when compared with HRSV B. While the literature does not report on an association between *S. pneumoniae* and HRSV subgroup‐specific infection, children hospitalized with HRSV infection and *S. pneumoniae* codetection in the nasopharynx have demonstrated worse clinical severity than those infected with HRSV only.^46^, ^47^, ^48^

We identified four circulating HRSV genotypes over the observation period, HRSV A: NA1 and ON1 and HRSV B: BA9 and BA10. Following global trends, the emergent HRSV A genotype, ON1, showed a gradual increase in prevalence from 5.6% (3/53) in 2012 to 81.5% (22/27) in 2015.^4^, ^8^, ^9^, ^10^, ^11^, ^12^, ^13^, ^49^ A complete replacement of the previously dominant HRSV A, NA1 genotype was not found as noted in several studies.^8^, ^11^, ^12^, ^13^ Three separate lineages of the ON1 genotype 1.1, 1.2, and 1.3 are circulating, and studies have suggested that the 1.1 lineage emerged prior to the other lineages. Circulation of the 1.1 lineage was also previously observed in South Africa in 2011–2012.^10^, ^12^ Here, we demonstrated the cocirculation of all three ON1 lineages in South Africa, as observed in most other countries that have conducted HRSV surveillance over two consecutive seasons since ON1 emergence.^12^ As with HRSV subgroups, prior studies have shown a spectrum of clinical outcomes associated with ON1 genotype infection.^4^, ^8^, ^15^, ^38^, ^44^ We found no association with clinical outcomes when comparing NA1 with ON1 genotypes. HRSV B genotype BA10 showed a gradual shift in prevalence from 33.3% (2/6) in 2012 to 78.6% (11/14) in 2015, albeit using limited study numbers.

This study was limited by only including individuals that were admitted to two sentinel SRI surveillance sites in the KwaZulu‐Natal and North‐West Provinces of South Africa. It is therefore possible that our study may not be an accurate representation of the general South African population or HRSV subgroup and genotype prevalence. In addition, only 23% (175/772) of the HRSV‐positive samples were genotyped. We therefore may not have shown the full diversity of HRSV genotypes circulating within the country. Furthermore, when we reviewed subgroup/genotype prevalence data available from other South African surveillance programs, the dominance patterns were the same (data not shown). Restricted study sampling may also have limited our ability to find statistically significant associations between measured variables and HRSV genotypes in all age groups and with HRSV subgroups in the ≥5‐year age group. It is therefore recommended that future studies in South Africa continue to follow HRSV prevalence and diversity trends in all age groups to ascertain the target populations for candidate vaccines. In these studies, a greater emphasis should be placed on sampling broadly across the population and genotyping a larger proportion of the HRSV‐positive samples.

## 5 CONCLUSION

This study builds upon our understanding of the clinical and epidemiological relevance of HRSV, its subgroups, and genotypes among patients hospitalized with SRI in South Africa. Changes in HRSV diversity over the study period demonstrated the presence and dominance of similar subgroups and genotypes found elsewhere in the world. This may suggest that no region‐specific considerations will be required when selecting appropriate vaccine target strains. Furthermore, we showed that the youngest age group (<6 months), which demonstrated the highest HRSV infection prevalence (47%) would benefit most from HRSV vaccine implementation, similar to findings from previous studies from South Africa (55%).^2^, ^50^ While no significant differences were found between the clinical outcomes and underlying illnesses associated with HRSV A and B infection or between HRSV A genotypes NA1 and ON1, a statistically significant association between HRSV A and *S. pneumoniae* coinfection in young children warrants further investigation of disease outcomes.

## DISCLAIMER

The findings and conclusions in this report are those of the author(s) and do not necessarily represent the official position of the funding agencies.

## CONFLICT OF INTEREST

Cheryl Cohen has received funding to her institution from Sanofi Pasteur, US Centers for Disease Control and Prevention, World Health Organization, and has received funding to attend a meeting from Parexel. Dr. Dawood reports personal fees from Pfizer‐South Africa and conference attendance sponsorship from MSD‐South Africa, Pfizer‐South Africa, and Biomiereux‐South Africa.

## AUTHOR CONTRIBUTIONS

**Ziyaad Valley‐Omar:** Conceptualization; data curation; formal analysis; investigation; methodology; project administration; resources. **Stefano Tempia:** Conceptualization; data curation; formal analysis; investigation; methodology; project administration; resources; supervision. **Orienka Hellferscee:** Conceptualization; data curation; methodology; project administration. **Sibongile Walaza:** Conceptualization; project administration. **Ebrahim Variava:** Conceptualization. **Halima Dawood:** Conceptualization; project administration. **Kathleen Kahn:** Conceptualization; project administration. **Meredith McMorrow:** Conceptualization; data curation; formal analysis; methodology; project administration; supervision. **Marthi Pretorius:** Conceptualization; methodology. **Senzo Mtshali:** Conceptualization; data curation; formal analysis; investigation; methodology; project administration. **Ernest Mamorobela:** Investigation. **Nicole Wolter:** Conceptualization; project administration. **Marietjie Venter:** Conceptualization; methodology; project administration; supervision. **Anne von Gottberg:** Conceptualization; investigation; project administration; supervision. **Cheryl Cohen:** Conceptualization; project administration; resources; supervision.

## PRESENTATION OF DATA

Data from this study were presented as a poster at the 11th International Respiratory Syncytial Virus Symposium, November 2018, Ashville, NC, USA (Abstract number: ARSVA0113).

### PEER REVIEW

The peer review history for this article is available at https://publons.com/publon/10.1111/irv.12905.

## Supporting information


**Table S1.** Factors associated with HRSV subgroups among patients aged ≥5 years, hospitalized with severe respiratory illness, Edendale and Klerksdorp‐Tshepong Hospitals, South Africa, 2012–2015.

**Figure S1.** Human respiratory syncytial virus (HRSV) seasonality in South Africa, 2012–2015. Bar graph showing monthly proportions of HRSV‐positive patients hospitalized with severe respiratory illness.

## Data Availability

Sequences with the following accession numbers MN516831 to MN517111 were uploaded to GenBank.
