# Supplementary material for: Human respiratory syncytial virus diversity and epidemiology among patients hospitalized with severe respiratory illness in South Africa, 2012–2015
Source: Influenza Other Respir Viruses. 2021 Sep 16;16(2):222–35. doi: 10.1111/irv.12905 (PMC8818822; doi:10.1111/irv.12905)
Supplement: Supplementary file 1 — Table S1. Factors associated with HRSV subgroups among patients aged ≥5 years, hospitalized with severe respiratory illness, Edendale and Klerksdorp‐Tshepong Hospitals, South Africa, 2012–2015. [file IRV-16-222-s002.docx]

**Supporting Table 1.** Factors associated with HRSV subgroups among patients aged ≥5 years, hospitalized with severe respiratory illness, Edendale and Klerksdorp-Tshepong Hospitals, South Africa, 2012-2015.

|  |  | HRSV A subgroup | HRSV B subgroup |  | |  |
| --- | --- | --- | --- | --- | --- | --- |
| Characteristic | **RSV subgrouped n/N (%)** | **Samples positive/ Samples tested (%)** | **Samples positive/ Samples tested (%)** | **Univariate Odds Ratio (95% CI)** | ***P* Value** | |
| Total | 70/171 (40.9) | 50/70 (71.4) | 20/70 (28.6) |  |  | |
|  |  |  |  |  |  | |
| Age |  |  |  |  |  | |
| 5-24 years | 18/70 (25.7) | 10/50 (20.0) | 8/20 (40.0) | Reference |  | |
| 25-44 years | 31/70 (44.3) | 26/50 (52.0) | 5/20 (25.0) | 4.2 (1.1-15.8) | 0.036 | |
| 45-64 years | 15/70 (21.4) | 9/50 (18.0) | 6/20 (30.0) | 1.2 (0.3-4.8) | 0.797 | |
| 65+ years | 6/70 (8.6) | 5/50 (10.0) | 1/20 (5.0) | 4 (0.4-41.5) | 0.246 | |
|  |  |  |  |  |  | |
| Female | 43/70 (61.4) | 33/50 (66.0) | 10/20 (50.0) | 1.9 (0.7-5.6) | 0.217 | |
|  |  |  |  |  |  | |
| Year |  |  |  |  |  | |
| 2012 | 19/70 (27.1) | 10/50 (20.0) | 9/20 (45.0) | Reference |  | |
| 2013 | 26/70 (37.1) | 24/50 (48.0) | 2/20 (10.0) | 10.8 (1.9-59.1) | 0.006 | |
| 2014 | 10/70 (14.3) | 4/50 (8.0) | 6/20 (30.0) | 0.6 (0.1-2.8) | 0.519 | |
| 2015 | 15/70 (21.4) | 12/50 (24.0) | 3/20 (15.0) | 3.6 (0.8-17.0) | 0.106 | |
|  |  |  |  |  |  | |
| Clinical presentation and course |  |  |  |  |  | |
| Fever ≥ 38^o^C | 29/69 (42.0) | 20/49 (40.8) | 9/20 (45.0) | 0.8 (0.3-2.4) | 0.750 | |
| Cough | 56/66 (84.8) | 40/48 (83.3) | 16/18 (88.9) | 0.6 (0.1-3.3) | 0.565 | |
| Supplemental oxygen needed | 35/69 (50.7) | 26/49 (53.1) | 9/20 (45.0) | 1.4 (0.5-3.9) | 0.543 | |
| Prolonged hospitalization (≥5 days) | 48/70 (68.6) | 36/50 (72.0) | 12/20 (60.0) | 1.7 (0.6-5.1) | 0.334 | |
| In-hospital death | 8/65 (12.3) | 8/45 (17.8) | 0/20 (0) | N/A | N/A | |
| Admitted to ICU | 69/69 (100) | 49/49 (100) | 20/20 (100) | N/A | N/A | |
| Tachypnea | Insufficient observations |  |  |  |  | |
| Stridor | Insufficient observations |  |  |  |  | |
|  |  |  |  |  |  | |
| Underlying medical conditions |  |  |  |  |  | |
| HIV infection | 54/68 (79.4) | 40/49 (81.6) | 14/19 (73.7) | 1.6 (0.5-5.5) | 0.475 | |
| Heart disease | 1/70 (1.43) | 1/50 (2.0) | 0/20 (0) | N/A | N/A | |
| Chronic lung disease | 70/70 (100) | 50/50 (100) | 20/20 (100) | N/A | N/A | |
| Asthma | 2/70 (2.9) | 2/50 (4.0) | 0/20 (0) | N/A | N/A | |
|  |  |  |  |  |  | |
| Co-infection |  |  |  |  |  | |
| Tuberculosis | 10/49 (20.4) | 7/35 (20) | 3/14 (21.4) | 0.9 (0.2-4.1) | 0.911 | |
| *Streptococcus pneumoniae* | 11/67 (16.4) | 8/47 (17.0) | 3/20 (15) | 1.2 (0.3-4.9) | 0.837 | |
| Influenza | 1/70 (1.4) | 1/50 (2) | 0/20 (0) | N/A | N/A | |
| Rhinovirus | 12/70 (17.1) | 8/50 (16) | 4/20 (20) | 0.8 (0.2-2.9) | 0.692 | |
| Adenovirus | 9/70 (12.9) | 5/50 (10) | 4/20 (20) | 0.4 (0.1-1.9) | 0.276 | |
| Enterovirus | 4/70 (5.7) | 3/50 (6) | 1/20 (5) | 1.2 (0.1-12.4) | 0.869 | |
| Human metapneumovirus | 1/70 (1.4) | 1/50 (2) | 0/20 (0) | N/A | N/A | |
| Parainfluenza virus types 1, 2, 3 | 70/70 (100) | 50/50 (100) | 20/20 (100) | N/A | N/A | |

N/A = OR omitted because of co-linearity, ICU = Intensive Care Unit, n/N = sample size/population size, CI = Confidence Interval
