# Supplementary material for: Human respiratory syncytial virus diversity and epidemiology among patients hospitalized with severe respiratory illness in South Africa, 2012–2015
Source: Influenza Other Respir Viruses. 2021 Sep 16;16(2):222–35. doi: 10.1111/irv.12905 (PMC8818822; doi:10.1111/irv.12905)
Supplement: Supplementary file 2 [file IRV-16-222-s001.docx]

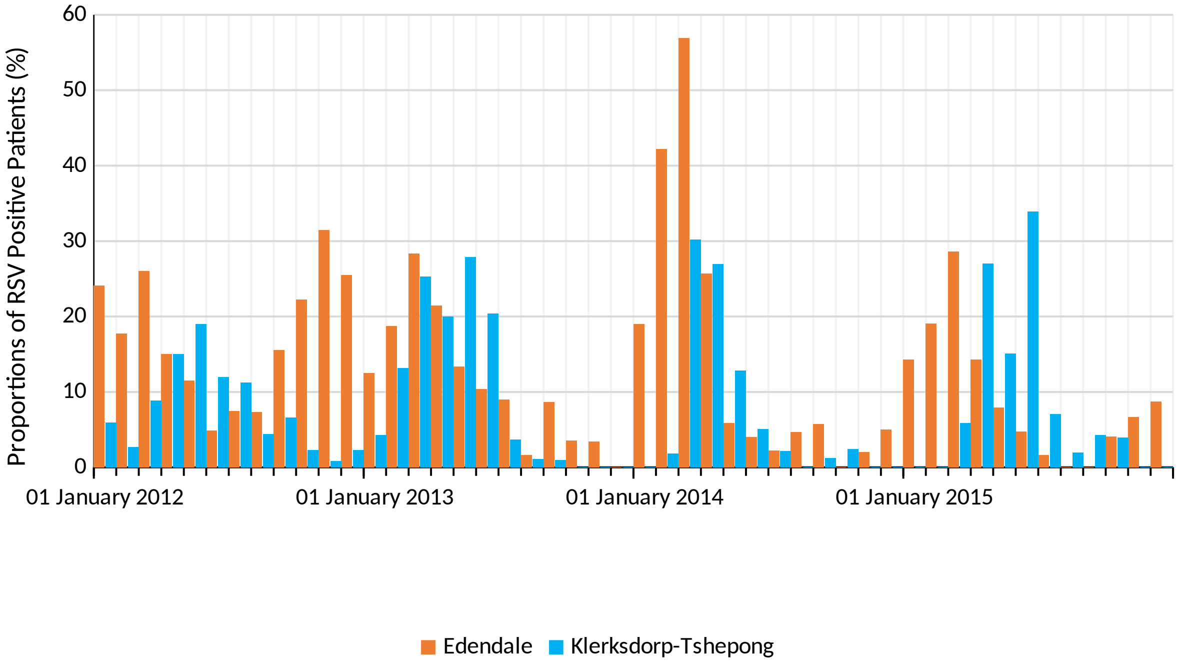

Proportions of RSV Positive Patients (%)
60
50
40
30
20
10
0
01 January 2012
01 January 2013
01 January 2014
01 January 2015
Edendale
Klerksdorp-Tshepong
